# IRDye800CW labeled uPAR-targeting peptide for fluorescence-guided glioblastoma surgery: Preclinical studies in orthotopic xenografts

**DOI:** 10.7150/thno.49787

**Published:** 2021-05-21

**Authors:** Sorel Kurbegovic, Karina Juhl, Kasper Kildegaard Sørensen, Julie Leth, Gro Linno Willemoe, Anders Christensen, Yvonne Adams, Anja Ramstedt Jensen, Christian von Buchwald, Jane Skjøth-Rasmussen, Michael Ploug, Knud J. Jensen, Andreas Kjaer

**Affiliations:** 1 Department of Clinical Physiology, Nuclear Medicine & PET and Cluster for Molecular Imaging, Department of Biomedical Sciences, Rigshospitalet and University of Copenhagen.; 2 Department of Chemistry, University of Copenhagen, Denmark.; 3 Finsen Laboratory and Biotech Research and Innovation Centre (BRIC), Rigshospitalet and University of Copenhagen, Denmark.; 4 Department of Pathology, Rigshospitalet, Copenhagen University Hospital, Denmark.; 5 Department of Otorhinolaryngology, Head and neck surgery & Audiology, Rigshospitalet, Copenhagen University Hospital, Denmark.; 6 Centre for Medical Parasitology, Department of Immunology and Microbiology, Faculty of Health and Medical Sciences, University of Copenhagen, Denmark.; 7 Department of Neurosurgery, Neuroscience Center, Rigshospitalet, Copenhagen University Hospital, Denmark.

**Keywords:** optical imaging, fluorescence-guided surgery, glioblastoma, uPAR, molecular targeted imaging

## Abstract

Glioblastoma (GBM) is a devastating cancer with basically no curative treatment. Even with aggressive treatment, the median survival is disappointing 14 months. Surgery remains the key treatment and the postoperative survival is determined by the extent of resection. Unfortunately, the invasive growth with irregular infiltrating margins complicates an optimal surgical resection. Precise intraoperative tumor visualization is therefore highly needed and molecular targeted near-infrared (NIR) fluorescence imaging potentially constitutes such a tool. The urokinase-type Plasminogen Activator Receptor (uPAR) is expressed in most solid cancers primarily at the invading front and the adjacent activated peritumoral stroma making it an attractive target for targeted fluorescence imaging. The purpose of this study was to develop and evaluate a new uPAR-targeted optical probe, IRDye800CW-AE344, for fluorescence guided surgery (FGS).

**Methods:** In the present study we characterized the fluorescent probe with regard to binding affinity, optical properties, and plasma stability. Further, *in vivo* imaging characterization was performed in nude mice with orthotopic human patient derived glioblastoma xenografts, and we performed head-to-head comparison within FGS between our probe and the traditional procedure using 5-ALA. Finally, the blood-brain barrier (BBB) penetration was characterized in a 3D BBB spheroid model.

**Results:** The probe effectively visualized GBM *in vivo* with a tumor-to-background ratio (TBR) above 4.5 between 1 to 12 h post injection and could be used for FGS of orthotopic human glioblastoma xenografts in mice where it was superior to 5-ALA. The probe showed a favorable safety profile with no evidence of any acute toxicity. Finally, the 3D BBB model showed uptake of the probe into the spheroids indicating that the probe crosses the BBB.

**Conclusion:** IRDye800CW-AE344 is a promising uPAR-targeted optical probe for FGS and a candidate for translation into human use.

## Introduction

Glioblastoma (GBM) is a severe cancer with basically no curative treatment and even with aggressive multimodal treatment the median survival is disappointing 14 months 1. Accordingly, multidisciplinary efforts are undertaken to understand the complex biology of the disease and to find better treatment modalities 2. Despite intensive research, only incremental improvements in GBM management and in overall survival have been accomplished within the last two decades. Surgery still remains the key treatment and while surgery cannot stand alone, the postoperative survival is determined by the extent of resection 3. The invasive growth with irregular infiltrating borders and islands of tumor cells embedded into the adjacent normal tissue complicates an optimal surgical resection with complete removal while at the same time sparing healthy brain tissue.

Usually, the anatomical location of the tumor is mapped preoperatively with computed tomography (CT) or magnetic resonance imaging (MRI) and integrated into an intraoperative neuro-navigation system. Expensive equipment, low spatiotemporal resolution, a time consuming and intricate workflow, and for CT, harmful ionization limits these modalities for continuous real time intraoperative imaging. Furthermore, intraoperative brain shift during neurosurgical procedures is well-known leading to a mismatch between the preoperative imaging and the actual intraoperative tumor location 4, 5. Although considered standard of care, MRI also cannot discriminate between pseudo and true progression or regression 6. In contrast, fluorescence imaging (FLI) enables *in vivo* visualization in real time with high spatiotemporal resolution without exposing subjects to harmful ionizing radiation. At present, 5-ALA, methylene blue 7, fluorescein 8, and indocyanine green (ICG) 9 are approved for human use by the Food and Drug Administration (FDA), but all lack cancer specificity. 5-ALA, methylene blue and fluorescein additionally suffers from short wavelength emissions in the visible spectrum and for 5-ALA rapid bleaching is also recognized 10, 11. These shortcomings limit signal intensity, tissue penetration, resolution and tumor-to-background ratio (TBR) due to nonspecific uptake in healthy tissue, autofluorescence, tissue absorbance, scattering, and photobleaching.

Better intraoperative imaging platforms for precise tumor visualization are therefore highly needed and molecular targeted near-infrared (NIR) FLI potentially constitutes such a tool. Ideally, targeting a molecular entity highly expressed by the cancer cells with a fluorescently labelled high affinity targeting molecule, could enable the cancer tissue to be specifically visualized with real-time FLI. This could guide the surgeon to distinguish cancerous tissue from healthy tissue for a complete resection of the tumor while preserving as much healthy tissue as possible.

The urokinase-type Plasminogen Activator Receptor (uPAR) belongs to the LU-domain protein family 12 and is expressed in most solid cancers including GBM 13, breast cancer, head and neck squamous cell carcinoma, pancreatic cancer, and lung cancer 14-18. uPAR expression is correlated to invasion and metastasis, one of the hallmarks of cancer, and the level of expression correlates to the aggressiveness of the cancer. Further, it is highly expressed at the invading front and the adjacent peritumoral activated stroma making it an attractive target allowing accurate margin visualization and tumor delineation 19.

Previously, we developed imaging probes that exploited the benefits of a small peptide (AE105) that specifically targets uPAR with high affinity 20. These comprised radionuclide labeled tracers for PET imaging 21-23 and fluorescently labeled probes for FLI with ICG (ICG-Glu-Glu-AE105) 24 and the novel NIR-II fluorophore CH1055 (CH1055-Glu-Glu-Glu-Glu-AE105) 25. Our previous optical probes were capable of visualizing orthotopic tongue cancer 26 and orthotopic pancreatic cancer 27 within the NIR-I window (750-900 nm) as well as orthotopic GBM through the intact skull of nude mice within the NIR-II window (1,000-1,700 nm). Although these probes did work well, further improvements and optimization may be possible, *e.g.,* obtaining higher TBR peaking at an earlier time point and remaining high for a longer time span.

In the present study, we therefore developed and tested *in vivo* a novel uPAR targeting probe using a new uPAR binding moiety in combination with the IRDye800CW fluorophore. The aim was to prepare for human translation by 1) improve TBR, pharmacokinetics, and signal intensity; 2) evaluate its cancer cell specificity using histology; and 3) establish a proof of concept for real-time fluorescence-guided resection of orthotopic human GBM xenografts in mice.

## Results

### Biochemistry, binding affinity, optical properties, and plasma stability

**Figure 1:**
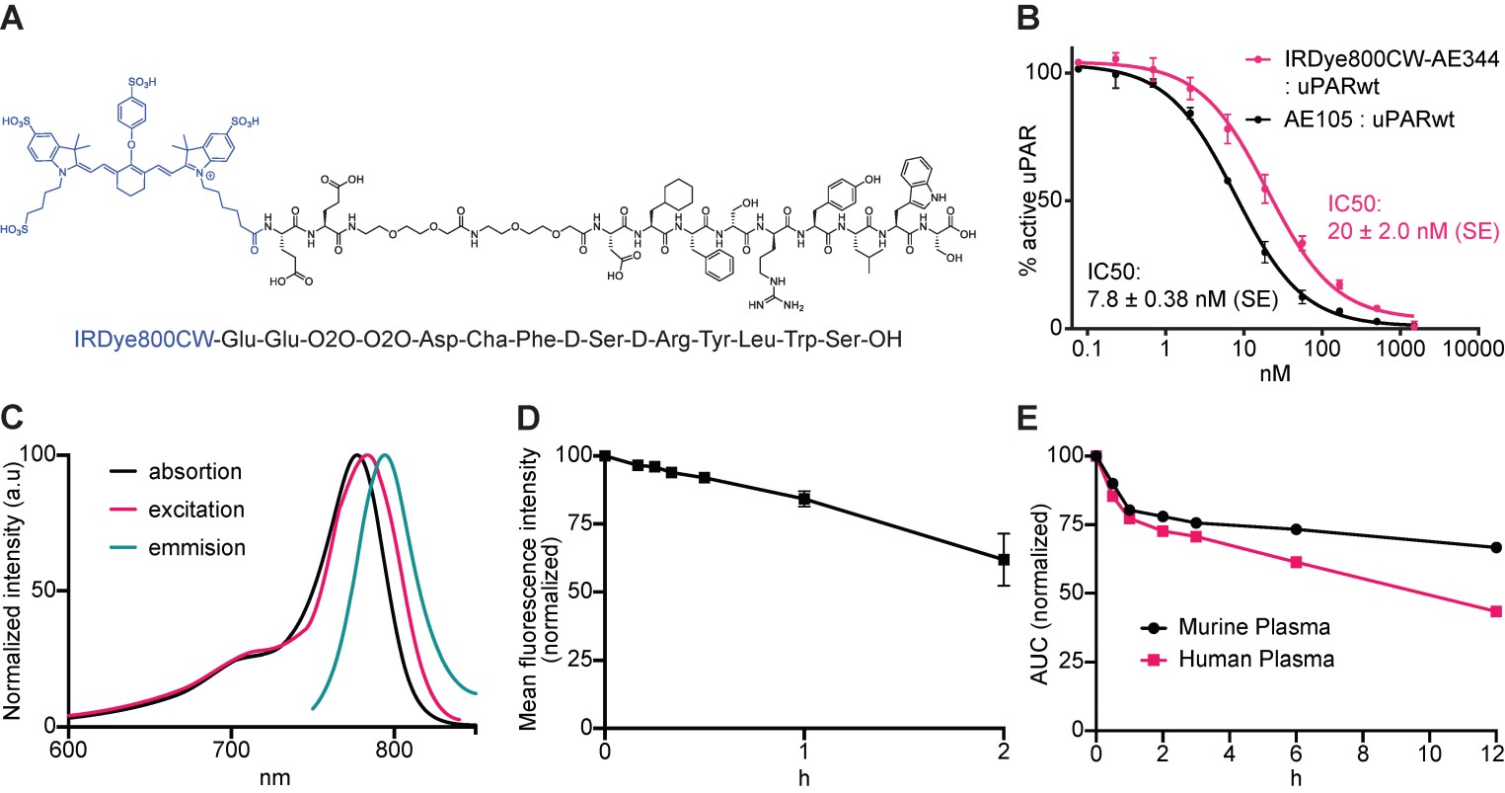
Biochemistry, binding affinity, optical properties, and plasma stability. **(A)** Molecular structure of IRDye800CW-AE344. **(B)** Surface Plasmon Resonance analysis of the affinity to uPAR. **(C)** Spectral analysis of the probe with absorption peak at 777 nm, excitation peak at 784 nm, and emission peak at 794 nm resulting in a Stokes shift of 10 nm. **(D)** Photostability of IRDye800CW-AE344 (normalized and pooled data from four different concentrations). **(E)** *Ex vivo* plasma stability quantified as area under curve (AUC) on HPLC (normalized to 0 h).

Building on our original uPAR-targeting peptide AE105, we designed a new peptide denoted AE344 with enhanced solubility due to the addition of a hydrophilic Glu-Glu-OEG_2_ linker at the N-terminal of AE105. This linker was chosen to spatially separate the positive charges in the targeting sequence from the negative charges of the NIR fluorophore IRDye800CW that was coupled to the N-terminal Glu (Figure 1A). The fluorophore-AE344 peptide was assembled by solid-phase peptide synthesis and subsequently purified by reverse-phase HPLC to 98% purity. As a negative control, we also synthesized a peptide, where the fluorophore was attached to the non-binding version of AE105, denoted AE354.

The ability to compete with binding of the natural ligand uPA to uPAR yielded an IC_50_ of 20 nM (SE = ±2.0 nM) as measured by surface plasmon resonance (Figure 1B), which is only slightly higher than the IC_50_ of 7.8 nM (SE = ±0.38 nM) measured for the competition of the unmodified uPAR-targeting peptide AE105.

The visible/NIR spectral properties was similar to free IRDye800CW with an absorption maximum at λ_abs,max_ = 777 nm (Figure 1C) and a slightly right shifted excitation profile with an excitation peak at λ_excit,max_ = 784 nm. The fluorescence emission spectrum showed peak emission at λ_emis,max_ = 794 nm resulting in a Stokes shift of 10 nm. Photostability revealed a preserved fluorescence intensity after continuous laser exposure of 84% for 1h and of 62% after 2h (Figure 1D).

The probe was stable within the relevant time window with normalized area under curve (AUC) values of 73% and 67% intact probe after 6 and 12 h, respectively in murine plasma, and 61% and 43% intact probe after 6 and 12 h, respectively in human plasma (Figure 1E).

### *In vivo* GBM imaging specificity

**Figure 2:**
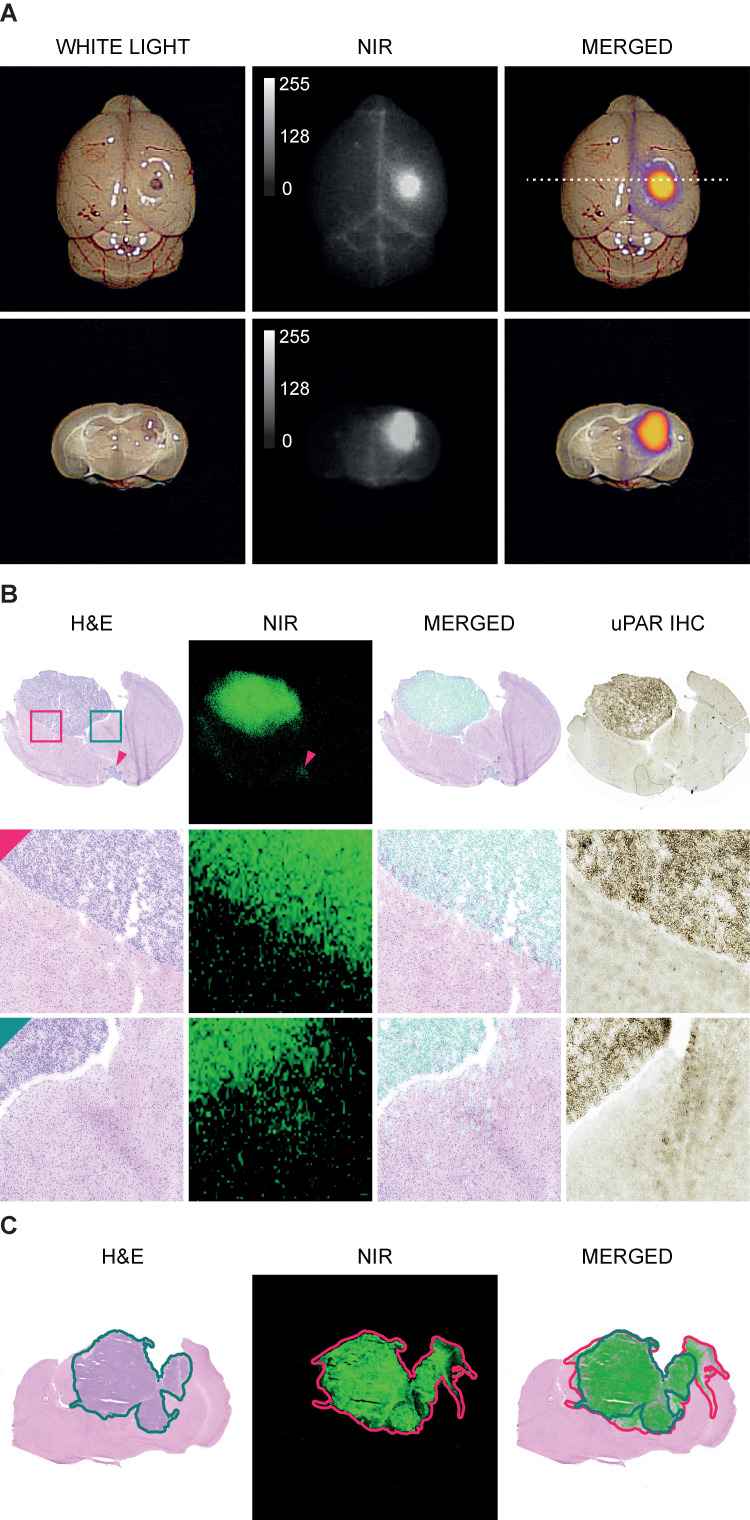
Imaging specificity.** (A)** Images acquired with EleVision™ IR (6 nmol IRDye800CW-AE344 at 1.5 h). Upper panel: Intact brain. Lower panel: Posterior part of the brain cross-sectioned corresponding to the dashed line in upper panel. **(B)** Histology of brain with H&E staining, NIR micro-imaging, H&E/NIR merged, and IHC staining of uPAR. The red arrow is pointing at a small leptomeningeal metastasis at the basis of the brain and is visible both on H&E staining and NIR microscopy (12 nmol, 24h). H&E staining and NIR micro-image are from the exact same slice while the IHC image is from the consecutive slices. **(C)** An illustration of an image pair used for colocalization between tumor on H&E and on NIR. Both images are from the same slide. The green line represents the tumor mask assessed on H&E and the red line represents the tumor mask on NIR. The overlap of the two masks is performed with Menders' coefficient. The example is illustrative by the NIR mask being larger than the H&E mask.

In white light (WL) the orthotopic GBM was not visible through the intact brain surface but was clearly visualized on NIR imaging 1.5 h following injection of 6 nmol IRDye800CW-AE344 (Figure 2A). Additionally, on cross-sectioned brain the tumor extent was visible with clear demarcation from healthy tissue allowing distinction between tumor tissue and healthy brain tissue. Histological assessment revealed co-localization of the tumor lesion on H&E staining, NIR micro-image with IRDye800CW-AE344, and uPAR immunohistochemistry (IHC) (Figure 2B). Menders' coefficients were 95.2 ± 3.7% (mean ± SEM) and 65.4 ± 7.2%, respectively for the fraction of tumor on H&E overlapping tumor on NIR, and fraction of tumor on NIR overlapping tumor on H&E.

### *In vivo* GBM imaging: Dose and time optimization

**Figure 3:**
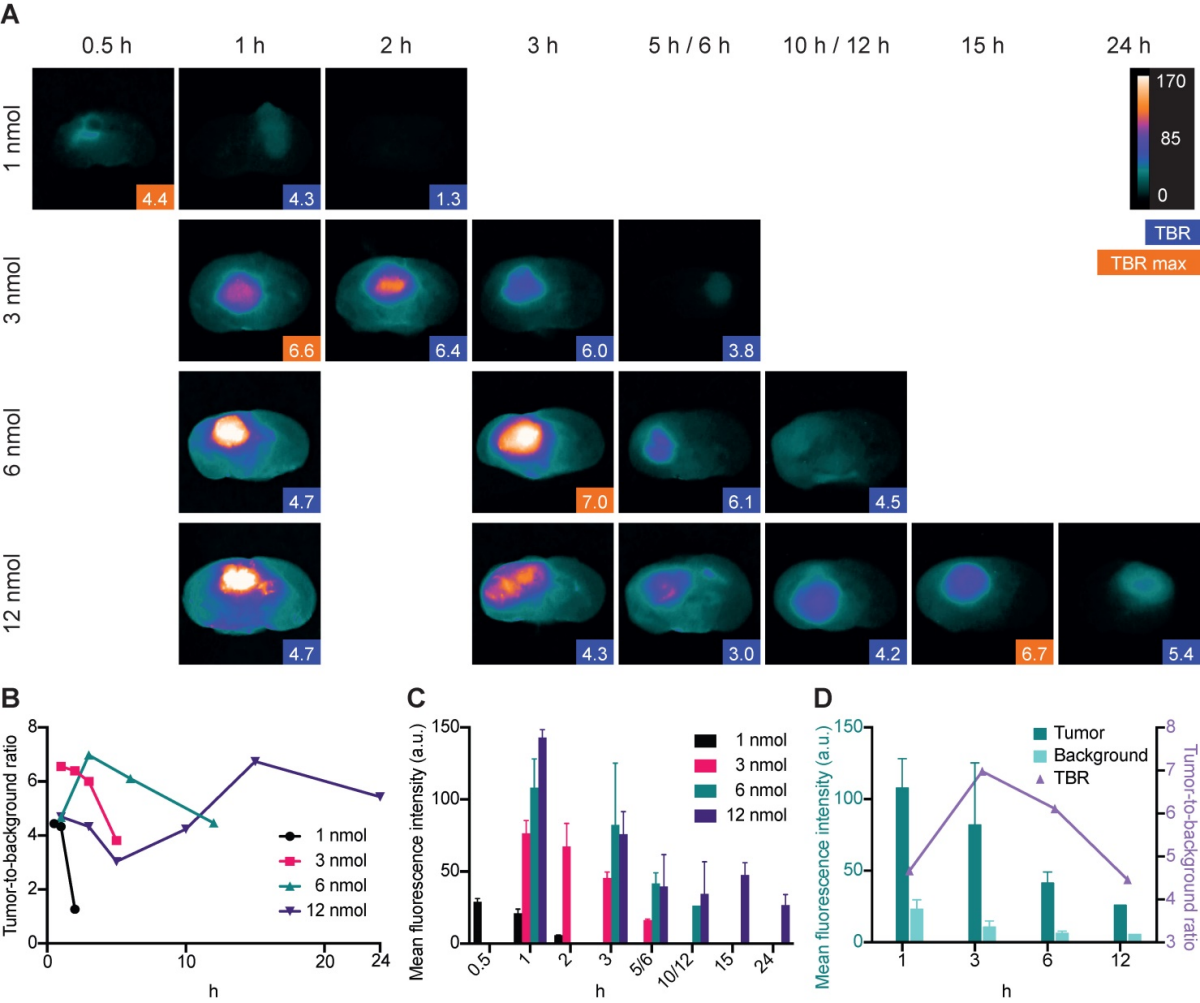
Dynamic imaging. All data are based on images taken with the Fluobeam with an exposure time of 333ms. **(A)** *Ex vivo* dynamic NIR imaging of GBM lesions on cross sectioned brains. Blue boxes indicate the TBR, and the orange boxes indicate the highest TBR for the given dose. Images are contrast enhanced equally with the scale bar representing the true values. **(B)** Tumor-to-background ratios for different doses of IRDye800CW-AE344. **(C)** Corresponding tumor mean fluorescence intensities. **(D)** Tumor and background mean fluorescence intensities at 6 nmol in comparison with the TBR. Tumor and background signal intensities are decreasing at different rates resulting in initial increase in TBR due to higher rate of background clearance. Subsequently, TBR is decreasing due to the opposite, higher tumor clearance compared to the background.

Imaging of orthotopic GBM lesions on cross sections revealed clear tumor visualization at four different IRDye800CW-AE344 doses (1, 3, 6 and 12 nmol). The highest TBR of 7.0 was observed 3 h after injection of a dose of 6 nmol (Figure 3A). At a dose of 1 nmol the maximal TBR of 4.4 was observed at 0.5 h, for 3 nmol the maximal TBR of 6.6 was observed at 1 h and for 12 nmol the maximal TBR of 6.7 was observed at 15 h (Figure 3B). The corresponding tumor mean fluorescence intensities (MFI) were 29, 77, 82 and 48 (a.u.) for 1, 3, 6 and 12 nmol doses, respectively (333 ms exposure time) (Figure 3C). MFI in both tumor and background were highest at the time of injection and decreased continuously over time. With 6 nmol the TBR initially increased from 4.7 at 1 h to 7.0 at 3 h followed by a decrease to 6.1 and 4.5 at 6 h and 12 h, respectively. At the same time there was a continuous decrease in tumor MFI with 108 (a.u.) at 1 h to 82 (a.u.) at 3 h corresponding to a 24% decrease (Figure 3D). The background MFI at the same timepoints decreased from 24 (a.u.) to 11 (a.u.) corresponding to a 54% decrease and the increase in TBR was thus a result of a higher background clearance rate compared to the tumor clearance rate between 1 h to 3 h.

### Fluorescence-guided resection

**Figure 4:**
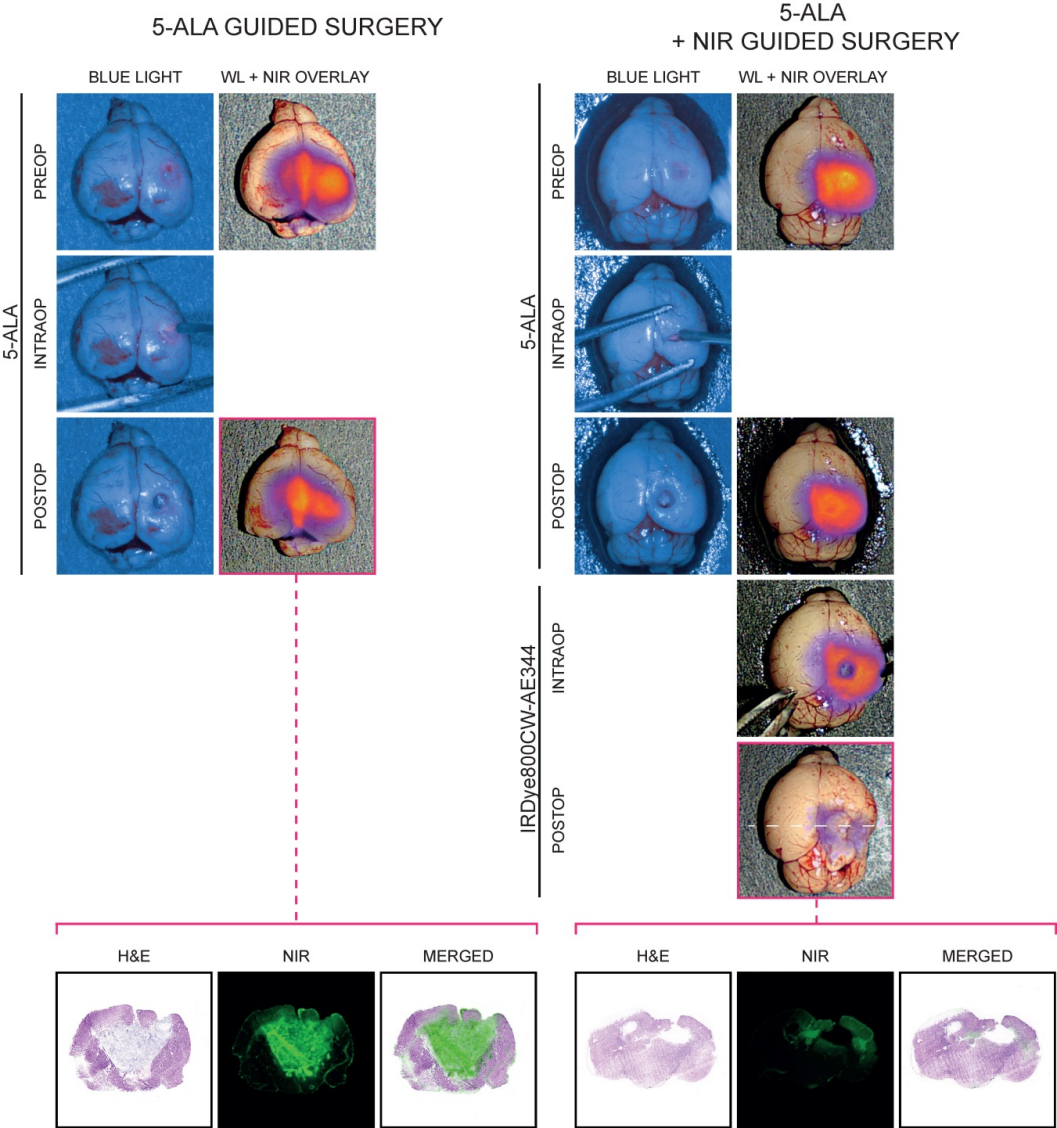
Fluorescence-guided surgery (IRDye800CW-AE344: 6 nmol at 3 h, 5-ALA: 0.25 mg/g body weight) performed in a combination of the EleVision™ IR and the ORBEYE systems on patient derived xenografts of GBM. Left panel: Surgery was guided by 5-ALA until the surgeon believed all 5-ALA signal was gone. The surgical bed was evaluated with NIR imaging (NIR images in third row) and corresponding histology of the postoperative residual tumor was performed clearly showing significant residual tumor (lowest row) (the visual impression that there's no resection cavity is an artifact due to collapse of the brain during tissue preparation. The brain should be as wide as right panel). Right panel: As left panel but following the 5-ALA surgery, the residual tumor was further resected guided by IRDye800CW-AE344 NIR imaging and corresponding histology of the NIR postoperative residual tumor was performed. It clearly shows decreased residual tumor, indicating that IRDye800CW-AE344 guided surgery is current setup is superior to 5-ALA.

Preoperatively, tumor was visualized with WL, blue light (BL) and NIR imaging and only with the NIR imaging with IRDye800CW-AE344 the tumor was convincingly visualized (Figure 4). *5-ALA guided surgery*: Intraoperatively, only when the tumor got exposed from overlying brain tissue, the 5-ALA signal became visible (Figure 4, left panel, intraop). When the surgeon believed that all 5-ALA positive tissue was resected, the surgical bed was evaluated by NIR imaging that revealed residual signal, indicating that not all tumor tissue was identified and removed with WL and 5-ALA (Figure 4, left, third row). The corresponding histological assessment clearly showed residual tumor supporting that the NIR fluorescence signal was in fact tumor. *5-ALA + NIR guided surgery*: Assisted by NIR, the surgeon identified additional tumor tissue and resected it until no or very little NIR signal was visible indicating complete tumor resection (Figure 4, right panel). The corresponding histological assessment clearly showed less residual tumor. A video of fluorescence-guided surgery is also available in the online supplementary materials.

### *In vivo* binding specificity

**Figure 5:**
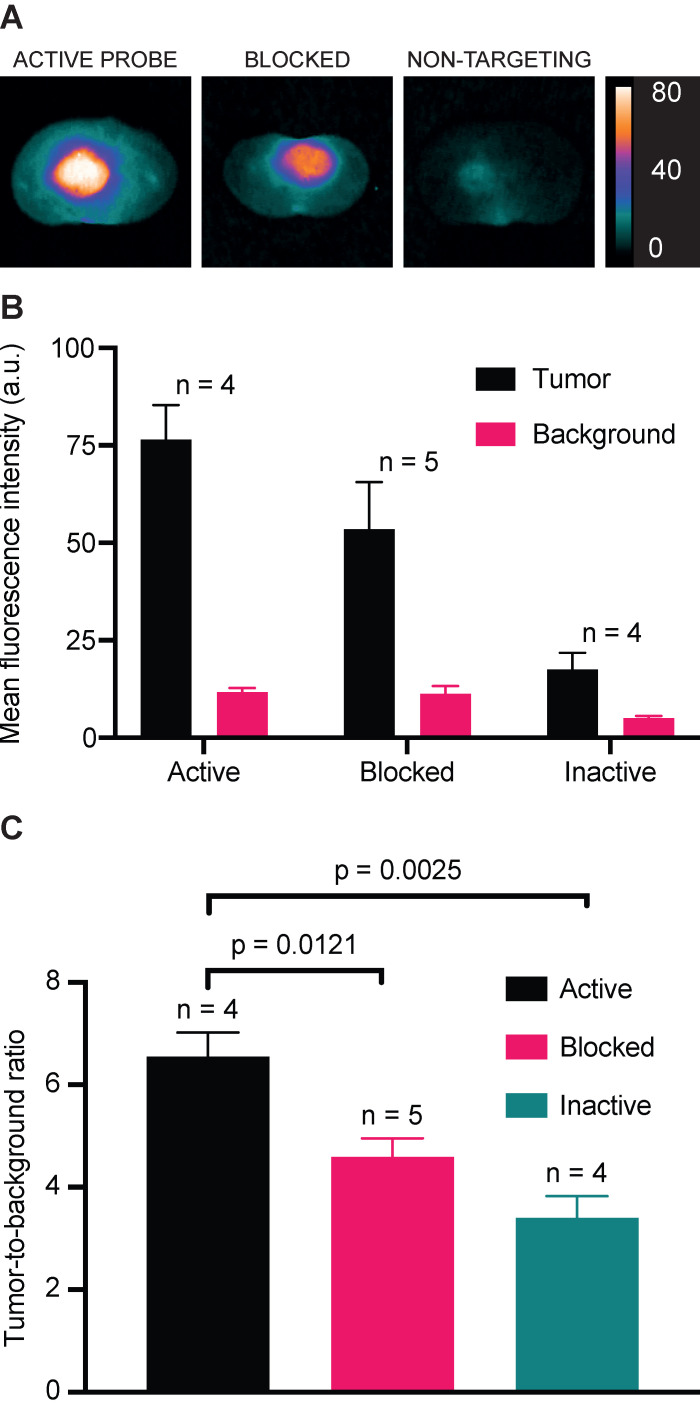
*In vivo* binding specificity. **(A)** NIR images of cross sectioned brains at 1 h after injection of (left to right): 3 nmol IRDye800CW-AE344 (active), 3 nmol IRDye800CW-AE344 + 1.7 mg AE120 (the dimer version of the AE105) (blocked), and 3 nmol IRDye800CW-*AE354* (binding inactive). Images are contrast enhanced equally with the scale bar representing the true values. **(B)** Mean fluorescence intensities for tumor and background. **(C)** The corresponding TBR values.

Competitive blocking of IRDye800CW-AE344 binding with AE120 (the dimer version of the AE105) and administration of the inactive non-targeting version IRDye800CW-*AE354* in orthotopic GBM showed lower signal intensity compared to the active probe (Figure 5A+B). The normalized TBR values for the groups receiving active, blocked, or inactive probe were 6.6, 4.6 (p = 0.012), and 3.4 (p = 0.0025), respectively (Figure 5C).

### Biodistribution and acute toxicity

**Figure 6:**
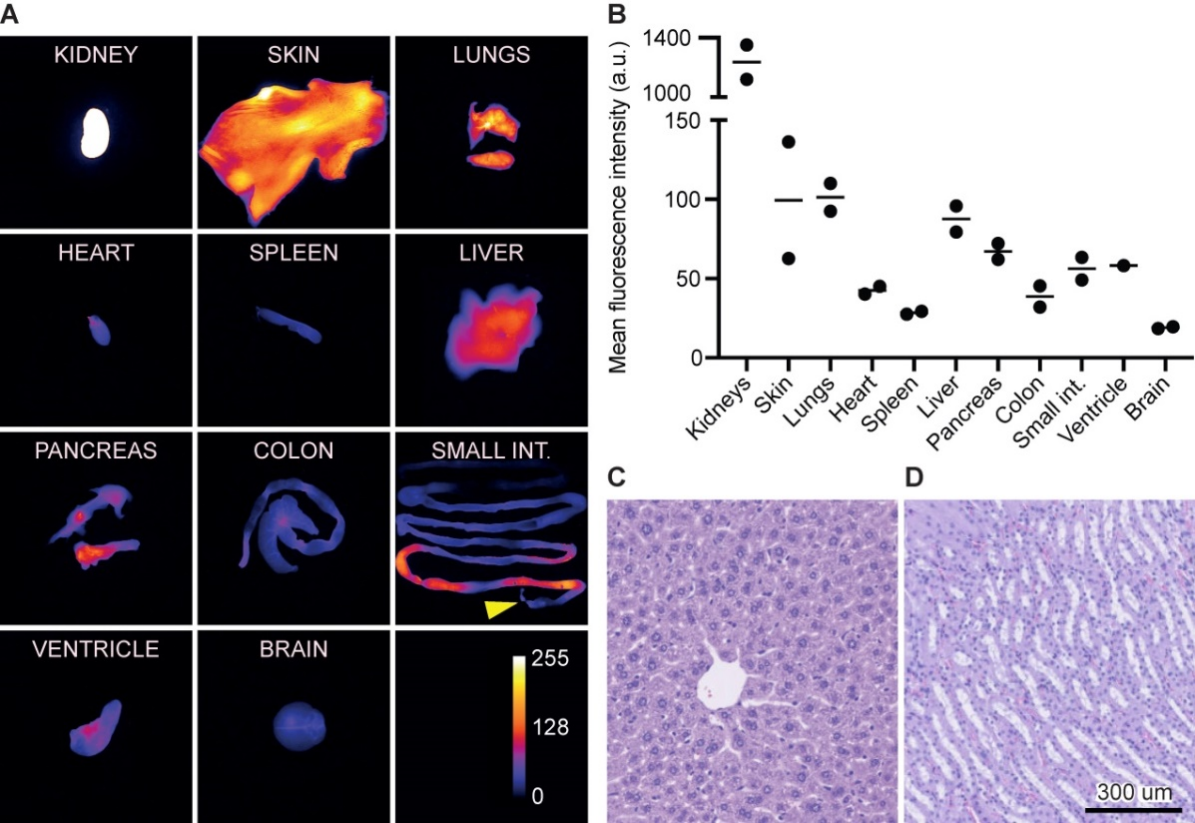
Biodistribution and acute toxicity.** (A)** Images of organs representing the fluorescence intensity and biodistribution of 3 nmol IRDye800CW-AE344 (1 h post injection, exposure time: 333ms). The yellow arrow at the small intestines indicates the proximal end. Kidneys are saturated and thus out of range of the color calibration bar.** (B)** Quantification of fluorescence signal from (A) (the data are normalized with the skin as the reference). **(C)** Liver histology (H&E stained) (24 h post injection of 12 nmol IRDye800CW-AE344). **(D)** Kidney histology (H&E stained) (24 h post injection of 12 nmol IRDye800CW-AE344).

At 1 h, the kidneys exhibited the highest MFI of 1,236 (a.u.) followed by the lungs, skin, and liver of 101 (a.u.), 99 (a.u.), and 88 (a.u.), respectively. In comparison, the normal brain tissue exhibited a signal of 19 (a.u.) (Figure 6A+B). The signal in the small intestine was limited to the proximal intestines/intestinal content and only modest signal was seen in the distal part.

Acute toxicity evaluation was performed on liver and kidneys 24 h post injection of 12 nmol. Histologically, the liver tissue was normal with lobular configuration without inflammation, fibrosis, cholestasis or deposits. The kidney tissue was normal with preserved glomeruli and tubules without atrophy, inflammation, or fibrosis (Figure 6C+D).

### Blood-brain barrier penetration

**Figure 7:**
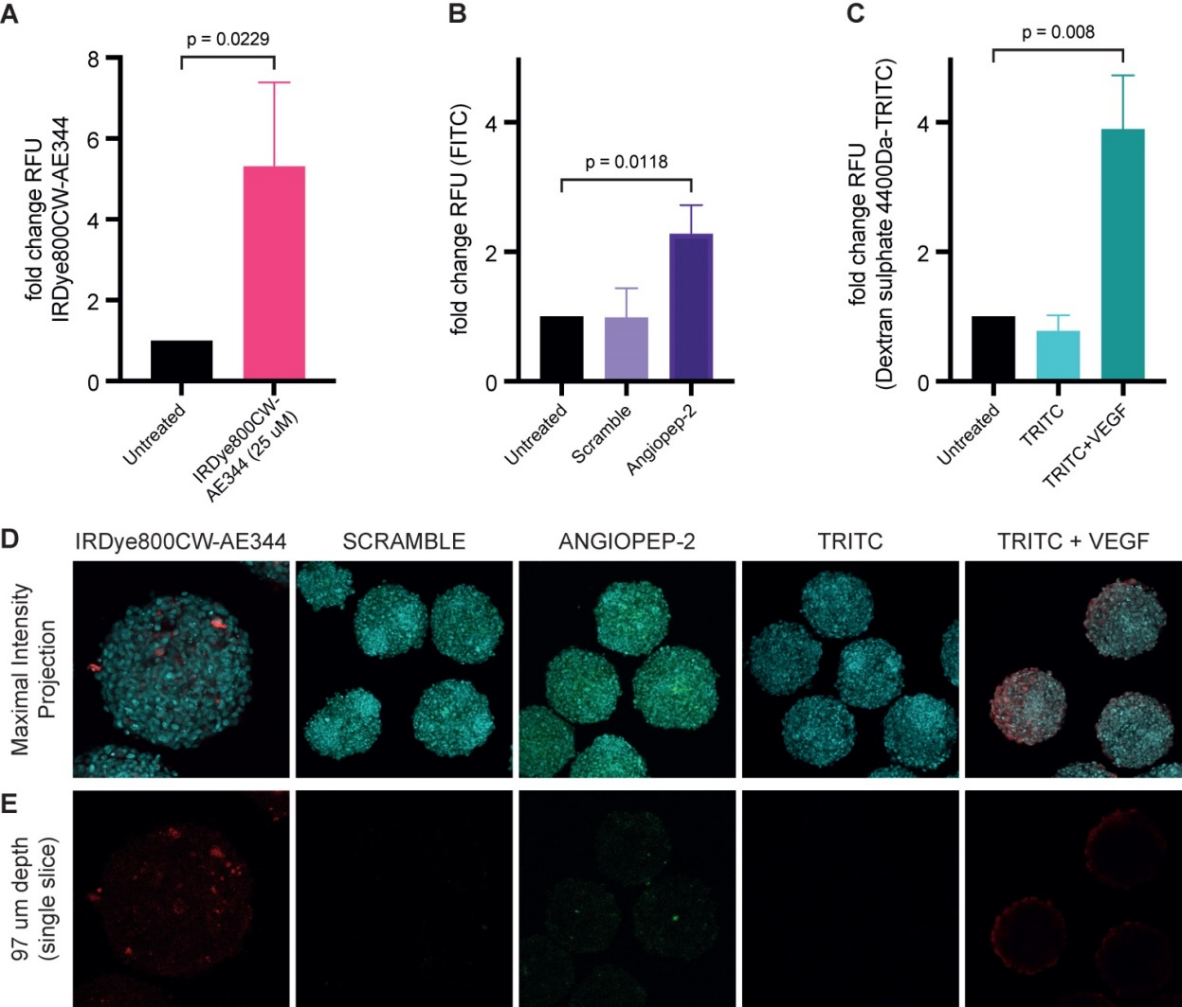
Measuring uptake of IRDye800CW-AE344 into 3D-Blood-Brain-Barrier (BBB) spheroids. Bar graphs showing **(A)** fold change in RFU in relation to uptake of IRDye800CW-AE344 (25µM). **(B)** Transport via transcytosis of angiopep-2 or scramble into BBB spheroids. **(C)** Verification of intact BBB by comparison of dextran sulphate 4.4kDa permeability with and without VEGF [50ng/µL] exposure. All values are compared to untreated controls. Statistical significance was determined using unpaired t-test *(A)* * = P 0.0229, whilst one-way ANOVA with Tukey's multiple comparison was performed on *(B)* * = P 0.0118, and *(C)* *** = P 0.008. Data represents three independent experiments, +/- SD. n^total spheroids^ = 466: n^assay 1^ = 145, n^assay 2^ = 139 and n^assay 3^ = 142. **(D)** Representative fluorescent confocal images showing maximum intensity projection of Z-stacks captured by Zeiss LSM 780, nuclei - cyan, IRDye800CW-AE344 - red, scramble and angiopep-2 - FITC/green, and dextran sulphate 4.4kDa-TRITC - red. **(E)** Representative confocal fluorescence images showing single slice corresponding to 97µm within the spheroids. Diffuse and localized staining pattern can be observed for IRDye800CW-AE344 (25µM), and Angiopep-2, (10µM) whilst VEGF exposure results in diffuse red/Dx 4-4kDa-TRITC (22.7µM) staining within spheroids. Both scramble (10µM) and Dx 4.4kDa-TRITC (22.7µM) failed to enter the spheroids. Scale bars = 100 µm.

Measuring uptake of IRDye800CW-AE344 (25 µM) into 3D blood-brain barrier (BBB) spheroids showed a 5.3-fold change in relative fluorescence units (RFU) compared to untreated spheroids (p = 0.0229) (Figure 7A+D+E). Transport via receptor mediated transcytosis of angiopep-2 or scramble into BBB spheroids showed a RFU of 2.8 *vs.* 0.99, respectively (p = 0.01) (Figure 7B+D+E). Verification of intact BBB was determined by the comparison of dextran sulphate 4.4kDa-TRITC permeability with and without VEGF [50ng/mL] exposure showed intact barrier with RFU of 3.9 with VEGF *vs.* 0.78 without VEGF (p = 0.0006) (Figure 7C+D+E). All values are compared to untreated controls (RFU=1.0).

## Discussion

Key features for an optimal fluorescent probe well-suited for surgical guidance are 1) high sensitivity and specificity, 2) high TBR achieved within few h, 3) need of a low dose to yield high fluorescence signal, and 4) a favorable safety profile. In the present study, we successfully synthesized a new peptide-based fluorescent probe that effectively visualized GBM *in vivo* with a TBR above 4.5 between 1 to 12 h after an injection dose of 6 nmol. This allows for flexible use and complies with the standard workflow at surgical departments where the probe can be injected shortly before surgery, *e.g.*, at the preparation for surgery or induction of anesthesia. The useful time-window starts when the surgery begins and persist throughout even long procedures. The use of a small peptide-based probe thus seems particularly applicable for clinical intraoperative imaging. Accordingly, we showed that we could perform fluorescence-guided resection of an orthotopic PDX in a mouse as early as 3 h after administration of 6 nmol IRDye800CW-AE344.

In contrast, the large molecular size of antibody-based probes impairs a high TBR at an early time point due to a long circulation time in the blood leading to a high background signal persisting for days. However, a high TBR can be obtained with antibody-based probes due to their usually very high affinity and although it can only be obtained by waiting days after injection it might persist for a longer time *e.g.*, days. An antibody-based fluorescent probe, IRDye800CW-Cetuximab, with a reported blood half-life of approximately 24 h has been tested in humans with GBM and showed feasible 28, 29. However, it needed to be infused 3 days prior to surgery. In our view, this may be impractical and does not comply with the established clinical workflow and requires the patient to come for an extra visit several days prior to the operation. Also, it is not uncommon that surgery is postponed or cancelled with short notice and in these cases, the antibody-based probe would already have been injected and thus be of no use. Injected antibodies, in general, also carries a well-known risk of side effects, especially immune-related adverse events 30. The use of antibody-based probes for fluorescence-guided surgery therefore seems inconvenient from a clinical perspective limiting their applicability and might not be the most ideal approach. Different strategies have been pursued to reduce the size while at the same time keep the high binding affinity properties of antibodies to improve the pharmacokinetic profile. This has resulted in different smaller antibody fragments, *e.g.*, nanobodies and affibodies, with high binding affinity and to some reduction in circulation time 31-33.

Compared to our previous probes, ICG-2Glu-AE105 and CH1055-4Glu-AE105, we improved several features. Importantly, the route of excretion changed from liver to kidneys leading to improved pharmacokinetics with faster excretion. Maximal TBR decreased from 15 h and 96 h of ICG-Glu-Glu-AE105 and CH1055-4Glu-AE105, respectively to 3 h with the current new probe. Furthermore, the affinity was increased with 7-fold (ICG-2Glu-AE105) and 4-fold (CH1055-4Glu-AE105) also adding to the increased TBR of 7.0 for the current probe compared to previous probes of TBR of 3.5 and 2.7, respectively.

We expect our new probe to be well tolerated and safe also in humans. This is based on the fact that its components have previously been used in humans with no safety issues. First, our binding moiety has previously been tested as a uPAR targeting PET probe (^68^Ga-NOTA-AE105) in multiple cancer types 14, 34, 35 (NCT03278275, NCT02755675, NCT03307460, NCT02960724, NCT02805608, NCT02964988, NCT02681640, NCT02965001) including an ongoing phase II trial in patients with GBM (NCT02945826). In total, more than 400 patients have so far been scanned and no adverse events or toxicity has been observed suggesting a favorable safety profile with no observed on or off target toxicity. Secondly, IRDye800CW is a novel fluorophore that has been tested safe in humans 28, 36-44, although not as extensively as the widely used fluorophore, ICG that has been proven safe on a larger scale. However, IRDye800CW has superior optical and pharmacokinetic properties with increased brightness, less bleaching and rapid renal clearance. Currently, at least 10 clinical trials have been performed with IRDye800CW labelled probes. Most of these studies are with IRDye800CW labeled antibodies 28, 36-43 and only one study has been with a labeled small peptide 44. There has been no reporting of severe adverse events and the reported mild side effects are most likely antibody reactions. On the basis of the safety profiles of the uPAR-targeting peptide and IRDye800CW, separately, it can be reasonably assumed that the safety profile of the compounds combined also exhibit a safe profile for human use. In support of this, our pathological assessment of the kidneys and the liver showed no pathological findings indicating no toxicity in these organs, especially the kidney where the probe accumulates and is cleared. Furthermore, IRDye800CW has spectral properties similar to ICG enabling compatibility with preexisting imaging equipment designed for ICG, which is key for a successful translation and clinical implementation.

Gliolan®, the brand name of 5-ALA, was approved for clinical use for GBM resection in Europe in 2007 and by the FDA in 2017. After oral administration, it is metabolized inside the tumor cells into protoporphyrin IX that is fluorescent with an emission peak at 635 nm. It was reported in a phase III clinical trial comparing 5-ALA and WL surgery that 5-ALA showed a significant improvement in progression free survival but not in overall survival 45. Further, the frequency of the most common early severe adverse events (hemiparesis, aphasia, convulsions, and epidural hematoma) was 11 of 139 patients in the 5-ALA group *vs.* 4 of 131 patients in the WL group but the rate of complete resection of the tumor increased from 36% to 65%. Therefore, 5-ALA has been widely adopted into GBM surgery. However, a recent COCHRANE review found low to very-low quality of evidence of 5-ALA improving glioma surgery 46 motivating new strategies for further improvement of GBM resection. Potentially, a more specific tumor delineation and visualization could minimize the risk of resection of non-tumorous tissue in the infiltrative zone of the tumor and further improve patient outcome.

On the optical side 5-ALA suffers some limitations compared to NIR as the excitation and emission spectra are within the visual spectrum. This overlap requires that the surgical light has to be BL in order to excite and allow visualization of the red emission light from 5-ALA limiting the optimal presentation of the anatomy for the surgeon during surgery. WL and excitation/emission of a NIR probe have distinct spectra allowing for complete separation of both excitation and emission light from WL. These optical properties give several advantages. The surgeon can use WL that optimizes the anatomical presentation of the tissue and at the same time use NIR imaging that can be merged onto the WL image as an overlay. Also, the separation of spectra means that the intensity of the WL and NIR can be adjusted separately. If the NIR fluorescence signal is too weak it can be increased separately from WL. NIR also allows for deeper visualization compared to 5-ALA that only visualizes the surface as both the excitation light and emission light in essence has no penetration through biological tissue. In present study we found that visualization was more convincing with NIR imaging with much higher signal intensity compared to 5-ALA and the 5-ALA signal was non-visible with overlying healthy brain tissue. Most importantly, following 5-ALA surgery the tumor resection was clearly incomplete with a surprising residual tumor load when evaluated postoperatively with NIR imaging and subsequent histology. Using NIR fluorescence guidance, we obtained a more complete resection with less NIR fluorescence signal that was validated by less tumor according to histology.

Accurate tumor visualization is important in neurosurgery and exact colocalization of fluorescence signal and tumor tissue is vital for FGS. We have quantified the co-localization between tumor on H&E slides and NIR fluorescence slides. Interestingly, the fraction of tumor on H&E overlapping tumor on NIR was 95.2% and higher than the fraction of tumor on NIR overlapping tumor on H&E of 65.4% meaning that the tumor area on NIR was larger than on H&E and, more importantly, that the NIR area included almost all tumor on H&E. uPAR is known to be highly expressed at the invading front of the tumor and the peritumoral activated stroma and this might be the explanation for the larger tumor area on NIR. Clinically it's relevant since it is exactly the infiltrative nature with single cell invasion into the peritumoral stroma that makes GBM difficult to resect completely. Being guided by NIR fluorescence might result in a maximal safe resection.

GBM as a high-grade glioma is characterized by alterations of the normal brain vasculature resulting in a disrupted leaky BBB 47 manifested on MRI with tumor contrast-enhancement. However, the highly invasive nature of GBM causes single cell invasion into otherwise healthy tissue and proliferation of cells in areas outside of the disrupted BBB region, usually in areas not showing contrast-enhancement on MRI 48. In contrast, the vasculature and BBB function in low-grade gliomas remains virtually unaltered and intact with normal function 49. As a consequence, the BBB in both high-grade and low-grade gliomas poses a major obstacle preventing sufficient drug delivery for effective treatment, contrast-enhanced imaging, and in this case molecular targeted fluorescence imaging 50. Interestingly, we found that our probe was able to internalize into 3D BBB spheroids indicating that the probe crosses the BBB effectively. Internal validation of the BBB model showed preserved function of the barrier with intact selective receptor mediated transcytosis further supporting that our probe truly crosses an intact and normal functioning BBB. This is interesting as it might result in more precise and accurate visualization of the GBM including tumor cells embedded in apparently healthy tissue where the BBB is intact. Furthermore, the probe might be able to visualize low-grade gliomas that are usually not possible to image with non-penetrating compounds. Accordingly, only approximately 20% of low-grade gliomas show visible fluorescence with 5-ALA 51, 52.

In the current study, proof-of-concept of FGS for GBM was obtained using a novel uPAR-targeting optical probe. This is an important first step towards successful translation into clinical FGS of GBM. However, it should be noted that the clinical setting is very different from that presented in our preclinical study. Imaging in humans leads to larger anatomical sizes and often the signal from the skin and other organs with high signal, *e.g.*, kidneys that may represent a challenge in a mouse model will be far away from the surgical field and therefore not be an issue. Also, imaging data and the procedural experience from a preclinical mouse model cannot be extrapolated directly to humans. Accordingly, further studies are needed to evaluate the full toxicological safety profile prior to human translation followed by efficacy and procedural safety testing in patients before implementation as a clinical routine.

## Materials and methods

### Biochemistry, binding affinity, optical properties, and plasma stability

A tumor targeting NIR probe was developed by conjugating the IRDye800CW fluorophore (LI-COR) with a small uPAR targeting peptide AE344. The non-binding peptide fluorophore-*AE354* was synthesized as a negative control.

The peptides were produced by Fmoc solid-phase peptide synthesis on an automated peptide synthesizer (Biotage® Syro I) using a Fmoc Ser(t-Bu) TentaGel S PHB 0.25 mmol/g resin. Subsequently, 5 mg of IRDye800CW was conjugated to the peptides by HATU/HOAt coupling.

The crude probe was purified by a 3-step process on RP-HPLC (on a Dionex Ultimate 3000 system with a fraction collector). *Step 1:* Preparative C18 column (Phenomenex Gemini, 110 Å 5 µm C18 particles, 21×100 mm), solvent A, water + 0.1% TFA, solvent B, acetonitrile + 0.1% TFA. Gradient elution (0-5 min: 5%; 5% to 60% 5-32 min) at flow rate 15 mL/min. The fractions containing the fluorescent probe were freeze dried. *Step 2:* Preparative C4 column (Phenomenex Jupiter, 300 Å 5 µm C18 particles, 21×100 mm), solvent A: water + 0.1% TFA, solvent B: Methanol + 0.1% TFA. Gradient elution (0-5 min: 5%; 5% to 60% 5-32 min) at flow rate 15 mL/min. The fractions containing the fluorescent probe were freeze dried. *Step 3:* Preparative C18 column (Phenomenex Gemini, 110 Å 5 µm C18 particles, 21×100 mm), solvent A: water + 0.1% TFA, solvent B: acetonitrile + 0.1% TFA. Gradient elution (0-5 min: 5% to 30; 30% to 40% 5-40 min) at flow rate 15 mL/min. The fractions containing the fluorescent probe were freeze dried. The identity of the peptides was confirmed by mass spectrometry and their purities were evaluated by 2-step analytical RP-HPLC.

IRDye800CW-AE344 was synthesized in 98% purity evaluated by 2-step RP-HPLC. The MS measured molecular weight (MW) was consistent with the MW: [M+2H]^2+^ 1380.0523; [M+3H]^3+^920.3723; [M+4H]^4+^ 690.5289 (expected mass: 2759.0835).

Surface plasmon resonance (SPR) was performed to determine the IC_50_-value of IRDye800CW-AE344 and unconjugated AE105 on the uPAR•uPA interaction in solution using a Biacore 3000™ instrument essentially as described 53. In brief; pro-uPA^S356A^ was immobilized on a CM5 sensor chip (immobilizing >5000 RU ~ 0.1 pmol pro-uPA/mm^2^) providing a very high surface density of pro-uPA. This results in a heavily mass transport limited reaction causing the observed association rates (ν_obs_) to be directly proportional to the concentrations of binding active uPAR in solution - given only low concentrations of uPAR are tested (here 0.06 nM to 2 nM). The analysis was carried out by measuring ν_obs_ of a fixed uPAR concentration (2 nM) incubated with a 3-fold dilution series of IRDye800CW-AE344 (ranging from 0.076 nM to 1.5 µM) for 300 sec at 20 °C with a flow rate of 50 µL/min. A standard curve was measured in parallel (2-fold dilution of uPAR covering 0.06 nM to 2 nM) including one repeated concentration point at the end to validate the biological integrity of the sensor chip. Running buffer contained 10 mM HEPES, 150 mM NaCl, 3 mM EDTA and 0.05% (v/v) surfactant P20, pH 7.4. The sensor chip was regenerated with two injections of 0.1 M acetic acid, 0.5 M NaCl. The parent peptide antagonist AE105 20 was analyzed in parallel as positive control.

Probe excitation and emission profiles were obtained in PBS with PTI QuantaMaster 400 (Horiba Ltd., Japan). Excitation profile was measured at λ_emission_ = 850 nm and the emission profile were measured at λ_excitation_ = 740 nm with xenon arc lamps as excitation source. Absorption was measured on a Cary 300 UV-Vis (Agilent, Santa Clara, CA, USA).

The photostability was evaluated by a factor 2 dilution series with four samples from 0.23-1.8 nmol IRDye800CW-AE344 in 100 µL phosphate buffered saline (PBS) placed in a black 96-well plate. The well was placed in a black box with the FLUOBEAM mounted in the top at a distance of approx. 20 cm from the well plate where the excitation irradiance is 5 ± 1 mW/cm^2^. Image acquisition was performed at 0 min, 10 min, 15 min, 20 min, 0.5 h, 1 h, 2 h, 3 h, 6 h, 10 h, 15 h, 24 h. Each dilution sample was normalized to time point 0 and the data from all four samples were pooled.

For plasma stability 1,600 ul human and murine plasma were separately incubated with 2.4 nmol IRDye800CW-AE344 in 10 ul PBS at 37 ºC in dark. 200 ul samples were collected at 0, 0.5, 1, 2, 3, 6, 12 and 24 h. Plasma proteins were precipitated by addition of 200 ul acetonitrile and the samples were centrifuged at 10,000 G for 10 min. The supernatant was collected for analysis. The supernatant from time zero from both the human and mouse serum was analyzed to establish the retention time of the intact IRDye800CW-AE344 on HPLC-MS on a RSLC Dionex Ultimate 3000 (Thermo) instrument coupled to a Bruker QTOF Impact HD II. The column was an Aeris widepore 3.6 µm C4 column (150 × 4.6 mm, Phenomenex) and the solvent system was solvent A: water containing 0.1% Formic acid; solvent B: acetonitrile containing 0.1% formic acid. Method: 0-1 min 5% solvent B, 1-18min 5%-50% solvent B with a flowrate of 1 mL/min. This showed that the intact molecule was eluded after 14 min. The method, column, and solvents were then transfer to another Dionex Ultimate 3000 (Thermo) which had a 3100-FLD fluorescent detector that employed 774 nm as excitation wavelength and measured the emission at 798 nm. All samples were then run using the settings above and the AUC at the 14 min peak was used to calculate the degradation with 0 h for both murine and human plasma as the reference.

### Cell line and PDX culturing

U-87 MG-*luc2* cells (Caliper, Hopkinton, MA, USA) were cultured in Dulbecco's Modified Eagle's medium (DMEM) + GlutaMAX added 10% fetal bovine serum and 1% Penicillin-Streptomycin at 37 °C in humid 5% CO_2_ air. The cells were passaged or harvested when reaching 80-90% confluency.

A subcutaneous ST610 PDX tumor from a parent animal was harvested for donor cells and the tumor was dissected into smaller pieces. Accutase (500 units/mL) and collagenase IV (400 units/mL) was added to enzymatically digested the tumor tissue to yield a single cell suspension. The process was stopped by adding growth media. The digest was filtered through a 100 µm filter, washed in PBS and resuspended in PBS to a final concentration of 600,000 cells/10 μL. The inoculation procedure is described above.

### Animal models

All experimental procedures in animals were carried out in accordance with the approval (2016-15-0201-00920) by The Animal Experiments Inspectorate, Denmark. 7-10-week-old female nude mice (strain: Rj:NMRI-Foxn1^nu/nu^, JANVIER LABS, France) were orthotopically xenografted with U-87 MG-*luc2* or PDX cells. Prior to any surgical procedure, the animals were anesthetized with Hypnorm (0.315 mg/ml fentanyl, 10 mg/ml fluanisone) + Midazolam (5 mg/ml) + sterile water in the ratio 1:1:2 by subcutaneous injection of 0.01 ml/g body weight of the solution. The orthotopic GBM tumor model was established by inoculation of 500,000 U-87 MG-*luc2* cells in 10 μL or 300,000 ST-610 PDX cells in 5 uL ice cold PBS in the right hemisphere (U-87 MG-*luc2*: 1.5 mm lateral and 0.5 mm posterior to the bregma at 2 mm depth, PDX: 1.8 mm lateral and 1.5 mm posterior to the bregma at 2 mm depth) using a stereotaxic frame (Kopf Instruments, Tujunga, CA, USA) with the automated microinjection pump UMP3 (WPI, Sarasota, FL, USA). The cells were injected with a flow rate of 45 nL/s with the syringe kept in place for further 3 min before retraction. Tumor growth was subsequently monitored on MRI with the BioSpec 7T (Bruker, Billerica, MA, USA) with axial and coronal T2-weighted sequences. When the tumor size reached 2-17 mm^3^, the animals were included in the fluorescence imaging protocol.

### Study design

Fluorescence imaging protocol: To characterize the *in vivo* biodistribution and tumor imaging properties, IRDye800CW-AE344 was administered through tail vein injection into all the GBM bearing mice (n = 35) at four different doses: 1 nmol, 3 nmol, 6 nmol, and 12 nmol. Animals were randomized into different doses and timepoints. Two animals were sacrificed for each time point and the brain was removed and cross sectioned through the tumor for imaging. The number of mice was chosen from an explorative perspective to obtain a trend of the TBR at different time point and doses while reducing the number of animals as possible (in accordance with the 3R principles of welfare in research animals). Preliminary testing revealed slower background clearance for the higher doses prolonging the time window. Accordingly, image acquisition was performed at following time points after injection:

- 1 nmol: 0.5 h, 1 h, and 2 h
- 3 nmol: 1 h, 2 h, 3 h, and 5 h
- 6 nmol: 1 h, 3 h, 6 h, and 12 h
- 12 nmol: 1 h, 3 h, 5 h, 10 h, 15 h, and 24 h

The target specificity to uPAR was evaluated by two different methods: (1) competitive blocking with co-administration of the high affinity uPAR targeting peptide AE120 (the dimer version of the AE105 20) and (2) IRDye800 conjugated to a uPAR-binding inactive peptide, *AE354* (IRDye800CW-*Glu-Glu-O2O-O2O-Asp-Cha-**Glu**-(D)Ser-(D)Arg-Tyr-Leu-**Glu**-Ser-OH*; amino acids in bold represent changes from the binding active IRDye800CW-AE344*)* with similar peptide length as the active peptide. The competitive blocking dose of 1.4-2.8 mg AE120 was administered by injection of 3 nmol IRDye800CW-AE344 and imaged at 1 h (n=5). The uPAR-binding inactive peptide was administered at 3 nmol and the animals were imaged after 1 h (n=4). The size of each group was prospectively chosen to be n=5 to allow for statistical analysis and since statistical significance was obtained with n=4 in two groups data collection was stopped.

Fluorescence guided surgery was carried out in 4 animals receiving 6 nmol IRDye800CW-AE344 and the animal equivalent dose of 5-ALA of 0,25 mg/g body weight. IRDye800CW-AE344 was administered intravenously 3 h prior to surgery and 5-ALA was administered by oral gavage of 200 uL 4-5 h prior to surgery. All mice had surgery performed guided by 5-ALA, two animals were sacrificed for histology and two animals had additional surgery guided by NIR imaging. Following NIR surgery the mice were sacrificed for histology.

Biodistribution was assessed in animals (n=2) receiving 3 nmol IRDye800CW-AE344 and was euthanized after 1 h for organ dissection and imaging (exposure time: 333 ms). Due to renal excretion, the fluorescence signals in the kidneys were out of scale compared to all the other organs. Thus, images were acquired at a lower exposure time of 40ms and the signal intensity was subsequently extrapolated to be comparable to the other organs.

Acute toxicity was evaluated on the liver and kidneys from 5 animals 24 h post injection of 12 nmol IRDye800CW-AE344.

### Blood-brain barrier penetration

Cell lines: Human primary astrocytes and pericytes were grown in astrocyte (AGM) and pericyte growth media (PGM) all purchased from Neuromics. The human cerebral microvascular endothelial cell line D3 (hCMEC/D3) was purchased from Cedarlane Labs and grown in Vascullife-Mv media (CellSystems). Astrocytes and pericytes were cultured on poly-L-lysine (2 µg/cm^2^) coated T25 flasks whilst hCMEC/D3 were cultured on collagen (rat tail type I, 50 µg/mL). All cell lines were incubated at 37 °C/5% CO_2_.

Blood-brain-barrier spheroid assembly and uptake assays: Blood-brain barrier (BBB) spheroids were assembled as previously described 54. Briefly, 50 µL of 1% agarose (w/v) in Milli-Q water is added to each well of low-binding 96 well plates (Thermo Fisher Scientific) and allowed to cool prior to the addition of cells. Confluent astrocytes, pericytes and hCMEC/D3 were lifted with 0.025% trypsin and resuspended in BBB-working media (Vasculife-Mv with VEGF omitted and 2% normal human serum). 1x10^3^ of each cell type is added sequentially to give a total volume of 100 µL per well. BBB-working media was added to each well to a final volume of 200 µL per well. Astrocytes were used between passage 2 and 4, pericytes between passage 4 and 6, and hCMEC/D3 between passages 29 and 30. Spheroids were allowed to self-assemble over 48-72 h in a humidified incubator (37 °C/5% CO_2_).

Barrier integrity, transcytosis and IRDye800CW-AE344 uptake assay: Barrier integrity was verified by incubating spheroids with TRITC-conjugated dextran sulphate (4.4 kDa, 22.7 µM) +/- VEGF [50 ng/mL], whilst receptor mediated transcytosis capabilities were confirmed by incubation with 10 µM FITC-conjugated Angiopep-2 (FITC-(Ahx)TFFYGGSRGKRNNFKTEEY-OH), or Scramble (FITC-(Ahx)FYNRGNTKYEFGFESRGKT-OH) overnight in a humidified incubator Uptake of 25µM IRDye800CW-AE344 was verified by overnight incubation in a humidified incubator (37 °C/5% CO_2_). The following day, spheroids (10 per treatment condition) were pooled by group into low-binding Eppendorf tubes (1.5 mL, Thermo Fisher Scientific) using 0.1% Triton X-100 treated pipette tips and washed 3 times with sterile PBS. Spheroids were then fixed with 3.7% formaldehyde (Sigma) for 10 min. Fixative was then washed away with sterile Milli-Q water, and spheroid nuclei are stained for 10 min with DAPI (330 nM). The spheroids were washed twice with sterile Milli-Q water to remove excess stain, and transferred to 8 well chamber slides (ibidi, glass bottom) for visualization and analysis with Zeiss LSM 780 confocal microscope. The extent of uptake into the spheroids was determined by capturing Z-stacks of each spheroid and measuring the integrated density of a region of interest 50µm from the outer boundary of the spheroid using a slice that corresponds to 97µm inside the spheroid 54. The integrated density was used to calculate the fold change in RFU between treated and untreated controls for the corresponding conditions and fluorophores. Data was expressed as fold change (RFU) +/- standard deviation and statistical significance is confirmed using unpaired t-test, or ANOVA with Tukey's multiple comparison test.

### Histopathology

Tumors: Histological assessment was used to evaluate the colocalization of the fluorescence signal and cancer cells. The cross sectioned brain specimens were cryostat sectioned for NIR micro imaging. Cryostat sectioning was performed by fixation of the specimen in Tissue-Tek O.C.T. on dry ice. The fixed tissue was sliced axially at 10 um thickness and mounted on slides for immediate fluorescence micro imaging with the Amersham Typhoon 5 (GE Healthcare, Chicago, Illinois, United States). Multiple slices were performed across the tumor with a distance of 50-100 um between every slice. Afterwards, the slices were either IHC stained with an in-house antibody, poly-rabbit-anti-human-uPAR, produced by Finsen Laboratory, Rigshospitalet (Copenhagen, Denmark) or H&E stained by common standard procedure. The stained slides were imaged with the ZEISS Axio Scan.Z1 slide scanner (Carl Zeiss, Oberkochen, Germany). Quantification of the colocalization of tumor on H&E staining and NIR was performed with the JACoP plugin for ImageJ using the Manders' coefficients method 55. The analysis was performed to a corresponding H&E and NIR image pair with 10 image pairs in total from 3 animals. On each image the tumor was delineated creating a mask representing the tumor and the fraction of tumor on H&E overlapping with NIR and the fraction of tumor on NIR overlapping with H&E was measured for each corresponding image pair.

Liver and kidney tissue was formalin fixed and paraffin embedded. Sections of 2-4 um thick were cut and a routine staining panel applied including: H&E, modified sirius, PAS and Masson trichome for both and additional PAS with silver for kidneys and PAS with diastase, iron, reticulin artisan and oxidized orcein for the livers. Livers were immunohistochemically stained, immunohistochemical evaluation on 3 µm thick sections was done using the CK7 antibody from Dako/Agilent, GA619 (clone OV-TL12/30) following the manufacturer's instructions. The staining took place on the Omnis from Agilent utilizing the EnVision Flex+ detection kit (GV800). The primary antibody was diluted using Antibody Diluent (Dako DM830) and were incubated for 20 min. The sections were counterstained with hematoxylin.

### Imaging systems

All image acquisition was performed as specified in the text with the commercially available Fluobeam (Fluosoft version: 2.2.1) (Fluoptics, Grenoble, France), the ORBEYE (Olympus Corporation, Shinjuku, Tokyo, Japan) and the EleVision™ IR (Medtronic, Minneapolis, MN, USA) with non-commercially available modifications. The EleVision™ IR system modifications were with a 785 nm excitation laser and an 800-850 nm band pass filter on the exoscope kindly provided by Alex Chanin and Karsten Bergh, Medtronic.

### Image processing and analysis

Images were analyzed and processed in ImageJ 1.52a, NIH, USA. Signal measurements were performed on the raw images from the Fluoptics system. Tumor signal was measured as an MFI of the whole tumor and background signal was measured as the mean of a representative area with no tumor on the contralateral hemisphere. Presented pictures are contrast enhanced in ImageJ with the function *Contrast Enhancement (Saturated pixels: 0.3%)*.

### Statistics

Statistics were performed in Prism version 8.1.0 (GraphPad, San Diego, CA, USA) using the unpaired Student's t-test (two-tailed) unless otherwise stated.

## Supplementary Material

Supplementary movies.

## Abbreviations

BBB: blood-brain barrier
BL: blue light
CT: computed tomography
FDA: Food and Drug Administration
FLI: fluorescence imaging
GBM: glioblastoma multiforme
ICG: indocyanine green
IHC: immunohistochemistry
MFI: mean fluorescence intensity
MRI: magnetic resonance imaging
NIR: near-infrared
RFU: relative fluorescence unit
SE: standard error
SEM: standard error of mean
TBR: tumor-to-background ratio
uPAR: urokinase-type Plasminogen Activator Receptor
WL: white light
